# Sustained low influenza vaccination in health care workers after H1N1 pandemic: a cross sectional study in an Italian health care setting for at-risk patients

**DOI:** 10.1186/s12879-015-1090-x

**Published:** 2015-08-12

**Authors:** Antonietta Giannattasio, Miriam Mariano, Roberto Romano, Fabrizia Chiatto, Ilaria Liguoro, Guglielmo Borgia, Alfredo Guarino, Andrea Lo Vecchio

**Affiliations:** Medicine and Health Sciences Department, University of Molise, Campobasso, Italy; Department of Translational Medical Sciences, Section of Pediatrics, University of Naples Federico II, Via S. Pansini, Naples, 80100 Italy; Clinical Medicine and Surgery Department, University of Naples Federico II, Naples, Italy

## Abstract

**Background:**

Despite consistent recommendations by all Public Health Authorities in support of annual influenza vaccination for at-risk categories, there is still a low uptake of influenza vaccine in these groups including health care workers (HCWs). Aim of this observational two-phase study was to estimate the immunization rates for influenza in four subsequent seasons and for pandemic H1N1 influenza in HCWs of a University Hospital, and to investigate its distribution pattern and the main determinants of immunization. Phase 1 data collection was performed in 2009–2010, during the peak of H1N1 pandemic. Phase 2 data collection, aimed to investigate seasonal influenza vaccination coverage in the three seasons after pandemic, was performed in 2012–2013.

**Methods:**

The overall H1N1 vaccination rate was derived by the Hospital immunization registry. In 2010, the personnel of three Departments (Infectious Diseases, Pediatrics and Gynecology/Obstetrics) completed a survey on influenza. A second-phase analysis was performed in 2012 to investigate influenza vaccination coverage in three consecutive seasons.

**Results:**

The first-phase survey showed a low coverage for influenza in all categories (17 %), with the lowest rate in nurses (8.1 %). A total of 37 % of health care workers received H1N1 vaccine, with the highest rate among physicians and the lowest in nurses. H1N1 vaccination was closely related to the Department, being higher in the Department of Infectious Diseases (53.7 %) and Pediatrics (42.4 %) than in Gynecology/Obstetrics (8.3 %). The second-phase survey showed the lowest rate of influenza vaccination in 2012/13 season. The main reasons for not being vaccinated were “Unsure of the efficacy of vaccine” and “Feel not at-risk of getting influenza or its complications”. Despite recommendations, influenza vaccine uptake remains poor.

**Conclusion:**

Immunization is largely perceived as a personal protection rather than a measure needed to prevent disease spreading to at-risk patients. Compulsory vaccination against influenza should be considered as a possible strategy, at least in health institutions where at-risk patients are admitted.

**Electronic supplementary material:**

The online version of this article (doi:10.1186/s12879-015-1090-x) contains supplementary material, which is available to authorized users.

## Background

The trust in medical institutions and perceived efficacy of official recommendations is central to the management of infectious diseases like influenza [1]. Each year, influenza continues to be an important source of morbidity and mortality, with a heavy impact on National Health Care System [2, 3]. Despite consistent recommendations by all Public Health Authorities in support of annual influenza vaccination for at-risk categories, there is still a low uptake of influenza vaccine in these groups including health care workers (HCWs) [4–6]. While influenza vaccination coverage in HCWs has progressively improved up to over 60 % in the 2010–2011 in U.S, in European Countries it continues to remain low and far below the European Centers for Disease Control and Prevention target objective of 75 % within 2014 [7, 8]. Recent evidence reported influenza vaccination rates ranging between 5.8 % (Greece 2006–07) and 35 % (Germany 2010–11) according to different influenza season and country [9, 10].

During a pandemic, effective initiatives supporting immunization are even more required by the Public Health Authorities. The experience with 2009 H1N1 pandemic influenza showed a substantial failure of public immunization [11]. H1N1 vaccination was actively and freely offered to all at-risk groups, but it was poorly accepted [11–14]. In Spain, where the seasonal vaccination coverage was 31 %, the rate recorded during the 2009 H1N1 pandemic season was father lower (22 %) [15].

Aim of this observational two-phase study was to estimate the immunization rates for influenza in four subsequent seasons and for H1N1 influenza in HCWs of a University Hospital, and to investigate its distribution pattern and the main determinants of immunization. Phase 1 data collection was performed in 2009–2010, during the peak of H1N1 pandemic. Phase 2 data collection, aimed to investigate seasonal influenza vaccination coverage in the three seasons after pandemic, was performed in 2012–2013.

## Methods

### Study design and setting

This cross-sectional study was carried out at a tertiary-care University Hospital in Southern Italy.

Before performing the survey, we calculated the rate of vaccination against H1N1 influenza among the HCWs of this University Hospital and in three Departments by using computerized H1N1 immunization records available at our institution in 2009.

Successively, a cross-sectional face-to-face survey was carried out in three Departments: Pediatrics, Infectious Diseases, and Gynecology/Obstetrics (OBGYN). This survey included two phases: in the phase 1, data on 2009 H1N1influenza vaccination and 2009–2010 seasonal influenza vaccination were collected between December 2009 and January 2010 in the three selected Departments; phase 2 was carried out in December 2012 and consisted in a retrospective evaluation of influenza vaccination coverage in three seasons (2010–2011, 2011–2012, 2012–2013) in the same Departments enrolled in phase 1. All staff physicians, residents, nurses, paramedics and administrative personnel were invited to participate to the survey for a total of about 400 HCWs in the phase 1. In phase 2, the number of potentially participating HCWs was lower than in 2009 (*n* = 352) because of the retirement of a percentage of employees, mainly in the group of nurses/paramedics.

### University Hospital H1N1 vaccination rate: computerized immunization records

According to the indications by the Italian Ministry of Health, 2009 H1N1 influenza vaccine was offered free of charge to all HCWs of the University Hospital in their workplaces. Immunization was actively encouraged by information campaign and other initiatives. A specific Local Pandemic Committee was established with the aim of informing about H1N1 infection, promoting vaccination and updating personnel on the course and risks of pandemic flu. To calculate the overall vaccination rate, the computerized immunization records provided by the Department of Human Resources of the University Hospital was related to the total number of HCWs employed in this University Hospital in 2009. Using these computerized data, H1N1 vaccine uptake was specifically analysed according to the health care category and according to the Department where HCWs worked (Pediatrics, Infectious Diseases, and OBGYN).

### Survey on attitudes on influenza immunization and vaccination coverage in HCWs of three University Departments

In the phase 1 of the survey, data on attitudes about 2009 H1N1 and seasonal influenza, vaccination uptake, and determinants of vaccination were collected through a questionnaire in three selected Departments (Pediatrics, Infectious Diseases, and OBGYN). We used an anonymous questionnaire consisting of a demographic section, professional category and 7 multiple-choice questions exploring determinants and barriers to influenza vaccination. Some questions required a yes/no response (e.g. “Did you receive the pandemic H1N1 flu vaccination?”). Additional questions pertained the vaccination status for seasonal influenza, reasons to be or not vaccinated with H1N1 vaccine, concern about acquiring seasonal influenza and H1N1 illness and the source of information about H1N1 influenza and vaccination. This questionnaire was prepared during the pandemic flu season by AG and ALV and it was previously submitted to a small group of residents and nurses of the Department of Pediatrics to test its clarity of presentation and reliability. Immediately after the peak of the pandemic flu, the questionnaire was administered for five consecutive days, starting from December 2009.

In the phase 2 of the study, performed in December 2012, a short version of the same questionnaire consisting of only 5-questions was again administered to HCWs of the same three Departments enrolled in phase 1, to evaluate influenza vaccination rates and main reasons to be or not vaccinated in three post-pandemic seasons (2010–2011, 2011–2012, and 2012–2013).

In both phases, the questionnaires were administered face-to-face.

This observational study was reviewed and approved by H1N1 Local Pandemic Committee of the University Hospital, designed and performed in accordance with the Declaration of Helsinki; ethical approval from a formal institutional review board was not required, according to local policies (http://www.comitatoeticofedericoii.it/vademecum). All the physicians enrolled, who reported their immunization status, signed a written informed consent.

### Statistical analysis

Influenza immunization rate was calculated as the proportion of vaccinated subjects to the total number of individuals in the categories studied. Immunization rate was calculated according to each Department and each professional category. Face-to-face interview avoided missing data.

Data are expressed as number and percentage or means ± standard deviations (SD), as appropriate. Comparison of categorical and continuous variables was performed using the Chi-square test and the Student t-test, respectively. When the study groups were more than 2, the ANOVA test was used for continuous variables and Chi-square for categorical variables. A p value <0.05 was considered statistically significant. A STROBE checklist for corss-sectional studies is available as additional material (Additional file 1).

## Results

### University Hospital H1N1 vaccination rate: computerized immunization records

According to data provided by the Department of Human Resources, in 2009 the overall number of HCWs of the University Hospital was 2557. According to data provided by the computerized immunization records, a total of 499 (19.5 %) HCWs received H1N1 vaccine. A comparative evaluation of H1N1 vaccination coverage in health care categories was as follows: 205/837 (24.5 %) staff physicians, 143/750 (19.1 %) residents, 144/868 (16.6 %) nurses and paramedics, 7/102 (6.9 %) laboratory and administrative personnel (*p* < 0.0001). When total data on H1N1 vaccination obtained by the computerized records were analysed in the three selected Departments, we found that H1N1 vaccine uptake was closely not only to the category of HCWs, but also to the belonging Department (Table 1). Based on data provided by the Department of Human Resources, the hospital workforce in 2009 consisted of 213 HCWs in the Department of Pediatrics, 59 in the Department of Infectious Diseases and 137 in the Department of OBGYN (total number: 409). Specifically, immunization rate was 50.8 % in Infectious Diseases Department, 40 % in Department of Pediatrics and 6.6 % in OBGYN (*p* < 0.0001).

**Table 1:** H1N1 vaccination rate in HCWs of three Departments according to data derived from the computerized immunization records of University Hospital

	Number of HCWs receiving H1N1 vaccination		
HCWs cathegory:	Dept. Pediatrics (*n* = 213)	Dept. Infectious Diseases (*n* = 59)	Dept. OBGYN (*n* = 137)
Physicians (*n* = 182)	56/95 (59 %)	15/22 (68.2 %)	9/65 (13.8 %)
Nurses/Paramedics (*n* = 185)	24/93 (26 %)	13/32 (40.6 %)	0/60
Others (*n* = 42)	5/25 (20 %)	2/5 (40 %)	0/12

HCW = health care workers

### First-phase survey: Pattern of influenza vaccination and determinants of vaccine uptake in three Departments

The phase 1 survey in the three selected Departments (Pediatrics, Infectious Diseases, OBGYN) was completed by 300/409 (73 %) HCWs (66 males; mean age 44.3 ± 12.6 years). The response rate varied according to the Department being 95 % in the Departments of Pediatrics, 91.5 % in the Infectious Diseases Department and 35 % in OBGYN. Overall, 132 participants were physicians (45 staff physicians, 87 residents), 136 were nurses or paramedics, and 32 were biologists or administrative personnel. Demographic data, opinion on the potential severity of influenza illness and influenza vaccination rates are provided in Table 2. Seasonal influenza and H1N1 influenza vaccines uptake in 2009 was really low and nurses were the least immunized category (Table 2). A close relation between H1N1 vaccination rate and the Department was found, being vaccine uptake significantly higher in Infectious Diseases (53.7 %) and Pediatrics (41.4 %) compared to OBGYN Department (4.1 %) (*p* < 0.0001). When divided in two groups according to age, H1N1 vaccination rate was significantly higher in HCWs aged ≤35 years (46/102, 45.1 %) than in those >35 years (67/198, 33.8 %; *p* = 0.04). No difference for seasonal vaccination rate between the two age-groups was found, being the rate very low in both groups.

**Table 2:** Demographic data, opinion regarding influenza and vaccination rates in 300 HCWs of three University Departments

	HCWs:				
	Staff physicians	Residents	Nurses	Others^a^	p
Number	45	87	136	32	
Males, n (%)	21 (46.7)	21 (24.1)	28 (20.6)	6 (18.7)	-
Mean age in years ± SD (range)	50 ± 9.3 (32–72)	28.7 ± 2.9 (25–44)	52.1 ± 7.4 (28–62)	43.1 ± 12.9 (24–60)	-
Are you afraid of H1N1 flu?, n (%)	19 (42.2)	11 (12.6)	49 (36)	13 (40.6)	0.0002
Are you more afraid of H1N1 flu than seasonal flu?, n (%)	24 (53.3)	16 (18.4)	39 (28.7)	12 (37.5)	0.0004
Which were the sources of information on H1N1 flu?, n (%)^b^					
- Television/newspapers	17 (37.8)	25 (28.7)	88 (64.7)	20 (62.5)	<0.0001
- Scientific web-sites	24 (53.3)	56 (64.4)	25 (18.4)	17 (53.1)	<0.0001
- Physicians of my hospital	27 (60)	58 (66.7)	68 (50)	18 (56.2)	NS
- My primary care provider	9 (20)	4 (4.6)	24 (17.6)	2 (6.2)	0.01
- Other sources	15 (33.3)	1 (1.1)	5 (3.7)	2 (6.2)	<0.0001
- No information	6 (13.3)	7 (8)	4 (2.9)	1 (3.1)	NS
Did you receive seasonal flu vaccination this year?, n (%)	13 (28.9)	19 (21.8)	11 (8.1)	8 (25)	0.001
Did you receive H1N1 flu vaccination?, n (%)	27 (60)	41 (47.1)	37 (27.2)	8 (25)	<0.0001
H1N1 vaccination rate in HCWs aged ≤35 years (%)	4 (14.8)	40 (97.6)	1 (2.7)	1 (12.5)	<0.0001
H1N1 vaccination rate in HCWs aged >35 years (%)	23 (85.2)	1 (2.4)	36 (97.3)	7 (87.5)	<0.0001

^a^Others include receptionists, clinical administrators and biologists

^b^More answers were possible

HCW = health care workers

**Fig. 1:**
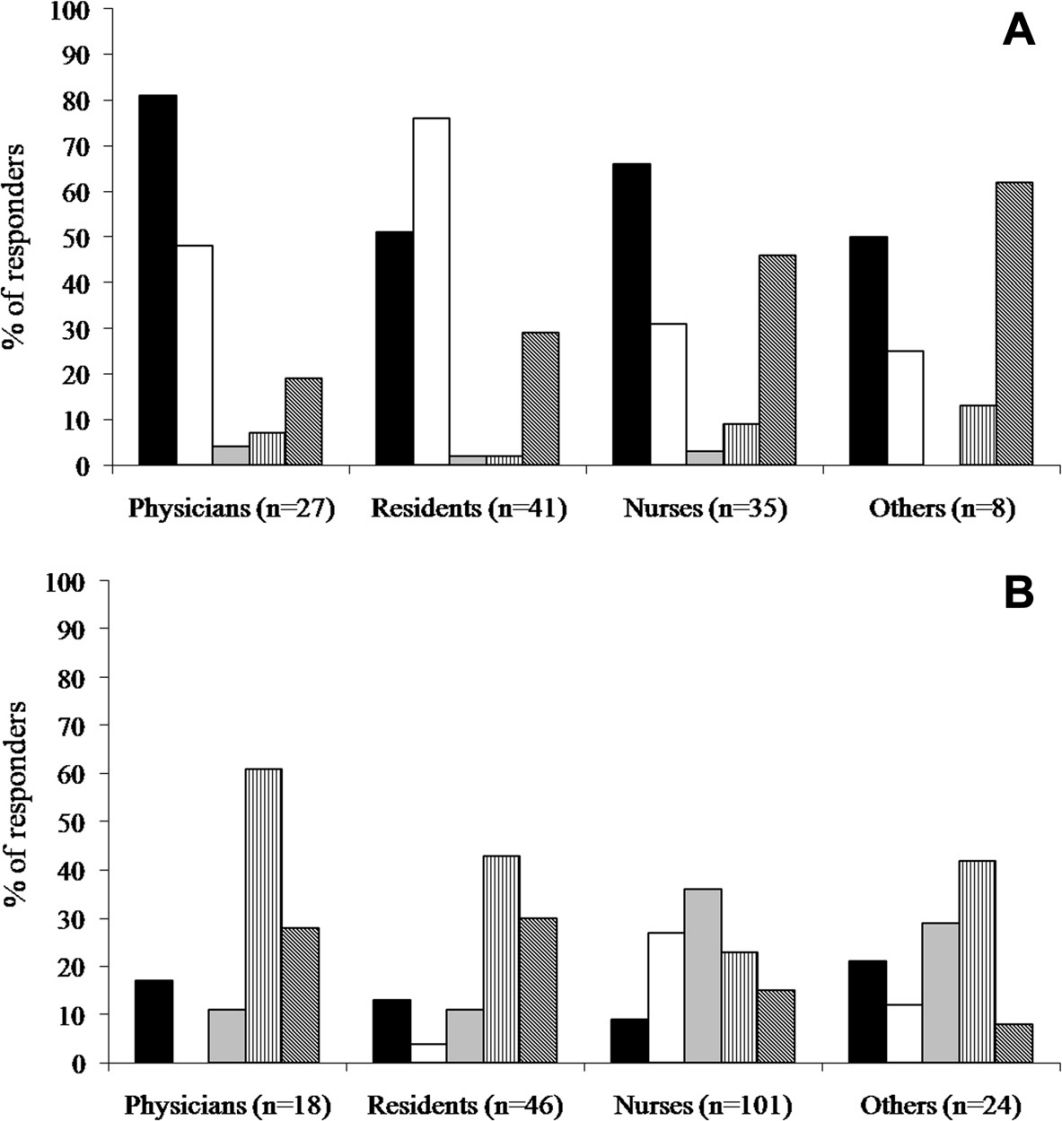
Reasons to be or not vaccinated against H1N1 influenza. **a**. Determinants of vaccination in 111 vaccinated HCWs. Response rate (%) to the question “Why did you get H1N1 influenza vaccination?” (more answers were possible). () Because I am a health care worker. () To protect my patients. () Because I am worried of H1N1 flu. () Because I have a chronic disease. () Other reasons. **b**. Reasons for missed vaccination in 189 non vaccinated HCWs. Response rate (%) to the question “Why did you miss H1N1 influenza vaccination?” (more answers were possible). Other reasons included: pregnancy (I trimester), previous allergy to seasonal influenza vaccine, H1N1 influenza diagnosed before H1N1 vaccine availability.() I am not afraid of H1N1 infection. () I am against vaccinations. () I am concerned about H1N1 vaccine side effects. () I am concerned about H1N1 vaccine efficacy. () Other reasons

Determinants of H1N1 vaccination are reported in Fig. 1. The main reasons for H1N1 vaccine uptake were “belonging to an at-risk category” (Fig. 1a). Reasons for not getting vaccination was scattered in different categories: lack of proof of “vaccine efficacy” was the main reason of concern in physicians, while the pattern of motivations against immunization was heterogeneous in nurses (Fig. 1b).

### Second-phase survey: Pattern of influenza vaccination and determinants of vaccine uptake in three Departments

A total of 206/352 (58.5 %) HCWs (49 males; mean age 41.3 ± 14.5 years) of the three Departments completed the second-phase survey. One-hundred and thirty-four participants were physicians (46 staff physicians, 88 residents) and 72 were nurses/paramedics. The lowest rate of influenza immunization was registered in 2012–2013 season, with a percentage of vaccinated HCWs of only 7.8 % (16/206), compared to about a 15 % of vaccinated employees in the previous two years (*p* = 0.03) (Table 3). In each season, influenza vaccination coverage was higher in physicians compared to residents and nurses (*p* < 0.05 in all cases). However, vaccination rate within each HCWs category differed according to the Department of origin (Table 4).

**Table 3:** Overall 2009 H1N1 vaccination rate and 2010-2011-2012 seasonal influenza vaccination rates in three University Departments

	Number of HCWs receiving influenza vaccination			
	Staff physicians (*n* = 46)	Residents (*n* = 88)	Nurses/paramedics (*n* = 72)	p
2009 H1N1 influenza	30 (65.2 %)	18 (20.4 %)	21 (29.1 %)	<0.0001
Seasonal 2010 influenza	17 (37 %)	10 (11.3 %)	4 (5.5 %)	<0.05
Seasonal 2011 influenza	16 (34.8 %)	8 (9 %)	8 (11.1 %)	<0.05
Seasonal 2012 influenza	8 (17.4 %)	4 (4.5 %)	4 (5.5 %)	<0.05
Never vaccinated against influenza	17 (37 %)	65 (73.8 %)	56 (77.8 %)	<0.0001

HCWs = health care workers

**Table 4:** 2009 H1N1 vaccination rate and 2010-2011-2012 seasonal influenza vaccination rates in three different University Departments

	Number of HCWs receiving influenza vaccination				
		Dept. Pediatrics (*n* = 144)	Dept. Infectious Diseases (*n* = 27)	Dept. OBGYN (*n* = 35)	Total (*n* = 206)
Vaccination 2009 H1N1	Physicians	25 (49 %)	2 (18.2 %)	3 (42.9 %)	69 (33.5 %)
	Residents	13 (25.5 %)	2 (18.2 %)	3 (42.9 %)	
	Nurses/Paramedics	13 (25.5 %)	7 (67.6 %)	1 (14.2 %)	
	Total	51 (35.4 %)	11 (40.7 %)	7 (20 %)	
Seasonal 2010 influenza	Physicians	13 (65 %)	2 (33.3 %)	2 (40 %)	31 (15 %)
	Residents	3 (15 %)	4 (66.7 %)	3 (60 %)	
	Nurses/Paramedics	4 (20 %)	-	-	
	Total	20 (13.9 %)	6 (22.2 %)	5 (14.3 %)	
Seasonal 2011 influenza	Physicians	12 (70.6 %)	2 (20 %)	2 (40 %)	32 (15.5 %)
	Residents	1 (5.9 %)	4 (40 %)	3 (60 %)	
	Nurses/Paramedics	4 (23.5 %)	4 (40 %)	-	
	Total	17 (11.8 %)	10 (37 %)	5 (14.3 %)	
Seasonal 2012 influenza	Physicians	6 (54.5 %)	1 (25 %)	1 (100 %)	16 (7.8 %)
	Residents	2 (18.2 %)	2 (50 %)	-	
	Nurses/Paramedics	3 (27.3 %)	1 (25 %)	-	
	Total	11 (7.6 %)	4 (14.8 %)	1 (2.9 %)	
Never vaccinated	Physicians	10 (10.1 %)	6 (50 %)	1 (3.7 %)	138 (67 %)
	Residents	48 (48.5 %)	3 (25 %)	14 (51.9 %)	
	Nurses/Paramedics	41 (41.4 %)	3 (25 %)	12 (44.4 %)	
	Total	99 (68.7 %)	12 (44.4 %)	27 (77.1 %)	

HCWs = health care workers

Reasons for being vaccinated for seasonal influenza included “I belong to a priority group for vaccination as HCW” (31.25 %), “To avoid infecting patients” (31.25 %), “Fear to be infected by influenza” (18.75 %), “To avoid infecting relatives” (12.5 %), and “I have a chronic disease” (6.25 %). No difference in response rates was found between physicians and nurses. Reasons for not receiving influenza vaccination were “Unsure of the efficacy of this vaccination” (33.7 %), “Feel not at-risk of getting influenza or its complications” (32.6 %), “Fear of this vaccine” (7.9 %), “I don’t believe in immunization” (7.4 %), or “Other reasons” (15.3 %). Reasons did not differ among employees’ category, with the exception of “I don’t believe in vaccines” (4.9 % in physicians versus 16.2 % in nurses, *p* = 0.02).

## Discussion

Our survey performed at a large tertiary-care University Hospital, where most patients are at increased risk for influenza because of underlying conditions, yielded worrying results considering the real low influenza vaccination coverage in a large sample of HCWs. This scenario may lead to relevant clinical consequences. It is to note that a substantial number of patients belonging to at-risk groups were admitted at our University Hospital because of H1N1 influenza indicating a massive number of chronic patients with the putative dangerous infection and the risk of spreading to patients with other risk conditions [16].

In our study, no significant increase in vaccination rates was observed during the 2009–2012 survey period. Indeed, the lowest prevalence of influenza vaccination was registered in this last season. Also during a pandemic, vaccination rate did not increase, and, although the rate was higher than for seasonal flu, H1N1 vaccination coverage was totally unsatisfactory.

In our study the vaccination rate appeared constantly low and stable during the 2009–2012 survey period. A trend in reduction was observed after the pandemic season and the lowest prevalence of influenza vaccination was registered in 2012–2013 season. Although data on the immunization rate before pandemic season is not available in out cohort, we could postulate that, after the initial fear related to pandemic, the coverage returned to a low pre-pandemic rate progressively moving far from the pandemic year.

Similar data come from other European and Italian series, with about 15-16 % of H1N1 immunization rate in HCWs [13, 17, 18].

Both general population and HCWs showed a low grade of acceptance of influenza vaccination due to public scepticism, low trust in health’ authorities recommendation, low perceived benefits of influenza vaccination for the individual and the community [11–14, 19]. Furthermore, personal reasons for not getting influenza vaccination include doubt about safety of vaccines, fear of adverse effects, feeling of not being at risk. and lack of suitable time or location for vaccination [20, 21]. In our study, concern regarding efficacy and safety of influenza vaccination was the main reason for not getting vaccination both for H1N1 and for seasonal influenza. Furthermore, H1N1 vaccination skepticism propagated through the media, ambiguous messages between activist groups against vaccination, Public Health Authorities, and Scientific Societies, and a delay in the distribution of vaccines, might have contributed to the widespread concern about this vaccination [7, 17]. Beliefs regarding vaccine efficacy in preventing HCWs infection and reducing influenza transmission to patients should be targeted through educational efforts to increase support for influenza vaccination.

We specifically analysed attitudes and barriers towards pandemic and seasonal influenza vaccinations. Physicians were more compliant with national recommendations for influenza immunization, while nurses were less keen to receive vaccination. Similar results have been reported in the other European and Italian surveys where the two major predictors of vaccination for H1N1 influenza were: type of occupation (being physician or, best, resident physician) and receiving seasonal influenza vaccine in the previous or current campaign [22]. This variability in vaccination rates among different HCWs has not been reported in U.S. where vaccination pattern did not differ between physicians, nurses and non-clinical staff [23]. Furthermore, we analysed vaccination coverage not only according the HCWs category but also the department of belonging. Department affiliation was the main determinant for getting immunization compared to work category. Such “Departmented” pattern of immunization, observed in conditions of a similar exposure to the pandemic vaccine and a similar pattern of at-risk patients, suggests that local practice and behaviours are involved strongly in immunization rate differences. The relatively high vaccination rate in the Departments of Pediatrics and Infectious Diseases has several explanations. Vaccination percentages were likely to be positively influenced by an increased sensitivity to the problem based on cultural background and by the activity of the H1N1 Local Pandemic Committee, mainly promoted by members of these two Departments. Although delivering women represented a category associated with a very high risk of severe outcome for H1N1, we surprisingly recorded the lowest vaccination coverage among staff members of the OBGYN Department. These are interesting findings to be considered in setting up immunization campaigns.

This study has some strength. Firstly, vaccination rate was calculated on the bases of face-to-face interviews. Secondly, it was analysed for 4-year consecutive periods. Most previous studies used their historical controls to compare the trend of immunization coverage or evaluated vaccination rate in only one season. Thirdly, the study was performed during and after the H1N1 pandemic alert, which allowed us to investigate both vaccinations.

Our findings may be considered representative of HCWs in other settings for several reasons: we investigated vaccination coverage in two medical Departments (Pediatrics and Infectious Diseases) and in one surgical Department (OBGYN). The first two had an active role in the building up of the H1N1 Local Pandemic Committee and in promoting H1N1 immunization. The involvement in the influenza vaccination campaign and the traditional attitude to infectious diseases prevention of pediatricians and infectivologists, compared to gynecologists, may partially justify the difference in response rates to the survey. Only a third of HCWs of OBGYN accepted to participate to the survey compared to almost all HCWs of Pediatrics and Infectious Diseases Departments, confirming a low interest of gynecologists in this topic.

Although data were not collected in the whole University Hospital, this method of sampling might have avoid, at least in part, participant selection bias. In contrast with other studies on influenza vaccination in HCWs including a strict age-range of respondents [24], age distribution of our sample was heterogeneous, thus the finding may be considered representative of the whole HCW population.

A limit of the study is represented by the non homogeneous sample of HCWs enrolled in the two-phases survey; this is related to the retirement rates of a high proportion of employees, mainly in the nurses group, in the last 3 years. Seasonal vaccination rates were essentially based on HCWs ‘recall and this may potentially results in a reporting bias; however, the information required were limited and easy to remember for each HCW.

Immunization data of HCWs not involved in the survey were not collected, however we reported that the immunization rates for H1N1 did not substantially differ between the computerized immunization record and the survey.

On the basis of this data, several findings should be considered in setting up immunization campaigns. Trust in recommendations on influenza vaccination should be improved among HCWs. by education and optimizing organizational barriers to allow flexible and workplace vaccine delivery. However, this approach alone may be not sufficient to improve vaccination rates considering that in our University Hospital H1N1 vaccination campaign combined education and promotion, free vaccination, flexibility of delivery of vaccine in terms of day of the week and hours, involvement of hospital’s leaders and administrative support.

Mandatory influenza vaccination for HCWs, especially in settings where high-risk groups are treated, should be considered as a possible strategy to increase vaccination rates. It has been reported that in U.S., in 2011–2012, influenza vaccination rate was 95 % among HCWs working in hospitals that required influenza vaccination, compared to 68 % among HCWs working in hospitals not requiring vaccination [25]. However, the attempt to legislate mandatory influenza vaccination for paramedics in the United States has met the opposition of HCWs who consider this policy as a violation of personal autonomy and choice [26]. A more feasible alternative to mandatory vaccination might be the written decline of vaccination by HCWs proposed in some settings [27, 28].

Anyway, to design adequate strategies to effectively implement vaccination is mandatory, mainly during a pandemic [29]. The H1N1 flu pandemic had limited impact, although not a negligible one, compared to the terrible scenarios envisioned in the preparedness plans. But, in truly disastrous pandemics and in conditions were the risk of exposure is high, compulsory vaccination should be considered for HCWs.

## Conclusions

Nevertheless all the efforts to increase influenza immunization coverage in HCWs, it remains on a low level. Information had a central role during regular and pandemic influenza. This paper shows that even in a supposedly best setting (teaching academic rather than regular hospital), in sensible departments (Infectious Diseases, Pediatrics, OBGYN), even when the heads of these departments were directly involved in promoting immunization, the overall effect in achieving flu immunization was far from being acceptable. In the light of the clinical risk associated with influenza in patients with chronic conditions, strategies and options alternative or adjunctive to those put forward for pandemic flu should be considered. We believe that immunization of HCWs operating in settings involving patients with high-risk conditions should be required as an ethical issue with clear consequences in terms of public health. Full immunization of HCWs should be a prerequisite without which any contact with patients should be permitted. We feel that the right of at-risk patients of being infecting with flu and other preventable infections is a clear priority.

## Additional file

Additional file 1:
**STROBE Checklist** (DOC 94 kb)

## Abbreviation

HCWs: Health care workers
OBGYN: Gynecology/Obstetrics
SD: Standard deviation

## References

[1] Gilles I, Bangerter A, Clémence A, Green EG, Krings F, Staerklé C (2011). Trust in medical organizations predicts pandemic (H1N1) 2009 vaccination behaviour and perceived efficacy of protection measures in the Swiss public. Eur J Epidemiol.

[2] Centers for Disease Control and Prevention (CDC) (2013). Update: Influenza activity - United States, September 30, 2012-February 9. MMWR.

[3] Giannattasio A, Lo Vecchio A, Napolitano C, Di Florio L, Guarino A (2014). A prospective study on ambulatory care provided by primary care pediatricians during influenza season. Ital J Pediatr.

[4] Giannattasio A, Lo Vecchio A, Franzese A, Prisco F, Femiano P, Guarino A (2010). Redundancy of roles by physicians in charge of paediatric diabetes is a barrier to flu immunisation. Arch Dis Child.

[5] Giannattasio A, Squeglia V, Lo Vecchio A, Russo MT, Barbarino A, Carlomagno R (2010). Pneumococcal and influenza vaccination rates and their determinants in children with chronic medical conditions. Ital J Pediatr.

[6] Pandolfi E, Carloni E, Marino MG, Atti ML C d, Gesualdo F, Romano M (2012). Immunization coverage and timeliness of vaccination in Italian children with chronic diseases. Vaccine.

[7] Del Campo MT, Miguel VJ, Susana C, Ana G, Gregoria L, Ignacio MF (2011). 2009–2010 seasonal and pandemic A (H1N1) influenza vaccination among healthcare workers. Vaccine.

[8] Centers for Disease Control and Prevention (CDC) (2012). Influenza vaccination coverage among health-care personnel: 2011–12 influenza season, United States. MMWR.

[9] Maltezou HC, Maragos A, Katerelos P, Paisi A, Karageorgou K, Papadimitriou T (2008). Influenza vaccination acceptance among health-care workers: a nationwide survey. Vaccine.

[10] Brandet C, Rabenau HF, Bornmann S, Gottschalk R, Wicker S (2011). The impact of the 2009 influenza A(H1N1) pandemic on attitudes of healthcare workers toward seasonal influenza vaccination 2010/11. Euro Surveill.

[11] Blasi F, Aliberti S, Mantero M, Centanni S (2012). Compliance with anti-H1N1 vaccine among healthcare workers and general population. Clin Microbiol Infect.

[12] European Centre for Disease Prevention and Control (2009). Use of specific pandemic influenza vaccines during the H1N1 2009 pandemic.

[13] Vírseda S, Restrepo MA, Arranz E, Magán-Tapia P, Fernández-Ruiz M, de la Cámara AG (2010). Seasonal and Pandemic A (H1N1) 2009 influenza vaccination coverage and attitudes among health-care workers in a Spanish University Hospital. Vaccine.

[14] Valour F, Bénet T, Chidiac C, Study group (2013). Pandemic A(H1N1)2009 influenza vaccination in Lyon University Hospitals, France: perception and attitudes of hospital workers. Vaccine.

[15] Sánchez-Payá J, Hernández-García I, García-Román V, Camargo-Angeles R, Barrenengoa-Sañudo J, Villanueva-Ruiz CO (2012). Influenza vaccination among healthcare personnel after pandemic influenza H1N1. Vaccine.

[16] Giannattasio A, Lo Vecchio A, Russo MT, Pirozzi MR, Barbarino A, Ruberto E (2010). Pandemic flu: a comparative evaluation of clinical, laboratory, and radiographic findings in HIV-positive and negative children. AIDS.

[17] Bangerter A, Krings F, Mouton A, Gilles I, Green EG, Clémence A (2012). Longitudinal investigation of public trust in institutions relative to the 2009 H1N1 pandemic in Switzerland. PLoS One.

[18] FluNews Weekly report 2010;21-March 15-21st: Website:http://www.epicentro.iss.it/focus/h1n1/pdf/flunews/FluNews_21.pdf Published 2010.

[19] Bouadma L, Barbier F, Biard L, Esposito-Farèse M, Le Corre B, Macrez A (2010). Personal decision-making criteria related to seasonal and pandemic A (H1N1) influenza-vaccination acceptance among French healthcare workers. PLoS One.

[20] Derber CJ, Shankaran S (2012). Health-care worker vaccination for influenza: strategies and controversies. Curr Infect Dis Rep.

[21] Hofmann F, Ferracin C, Marsh G, Dumas R (2006). Influenza vaccination of healthcare workers: a literature review of attitudes and beliefs. Infection.

[22] Esposito S, Bosis S, Pelucchi C, Tremolati E, Sabatini C, Semino M (2008). Influenza vaccination among healthcare workers in a multidisciplinary University hospital in Italy. BMC Public Health.

[23] Centers for Disease Control and Prevention (CDC) (2010). Interim Results: Influenza A (H1N1) 2009 Monovalent and Seasonal Vaccination Coverage Among Health-Care Personnel-United States, August 2009-January 2010. MMWR.

[24] Banach DB, Zhang C, Factor SH, Calfee DP (2013). Support for mandatory health care worker influenza vaccination among allied health professionals, technical staff, and medical students. Am J Infect Control.

[25] Centers for Disease Control and Prevention (CDC) (2011). Influenza vaccination coverage among health-care personnel, United States, 2010–2011 influenza season. MMWR.

[26] Lei Y, Pereira JA, Quach S, Bettinger JA, Kwong JC, Corace K (2015). Examining Perceptions about Mandatory Influenza Vaccination of Healthcare Workers through Online Comments on News Stories. PLoS One.

[27] Polgreen PM, Septimus EJ, Parry MF, Beekmann SE, Cavanaugh JE, Srinivasan A (2008). Relationship of influenza vaccination declination statements and influenza vaccination rates for health care workers in 22 US hospitals. Infect Control Hosp Epidemiol.

[28] Ribner BS, Hall C, Steinberg JP, Bornstein WA, Chakkalakal R, Emamifar A (2008). Use of mandatory declination form in a program for influenza vaccination of healthcare workers. Infect Control and Hosp Epidemiol.

[29] Giacomet V, Tarallo L, De Marco G, Giannattasio A, Barbarino A, Guarino A (2007). Preparing fora n influenza pandemic in Italy: resources and procedures in paediatric hospital units. Euro Surveill.

